# Optical Coherence Tomography Analysis of Retinal Layers in Celiac Disease

**DOI:** 10.3390/jcm11164727

**Published:** 2022-08-12

**Authors:** Livio Vitiello, Maddalena De Bernardo, Luca Erra, Federico Della Rocca, Nicola Rosa, Carolina Ciacci

**Affiliations:** 1 Eye Unit, Department of Medicine, Surgery and Dentistry, “Scuola Medica Salernitana”, University of Salerno, 84081 Salerno, Italy; 2 Celiac Centre at University Hospital San Giovanni Di Dio e Ruggi d’Aragona, University of Salerno, 84084 Salerno, Italy

**Keywords:** celiac disease, OCT, optical coherence tomography, retinal layers, RNFL

## Abstract

Celiac disease is an immune-mediated, chronic, inflammatory, and systemic illness which could affect the eye. The aim of this study is to look for possible signs of retinal involvement in celiac disease that could be utilized as biomarkers for this disease. Sixty-six patients with celiac disease and sixty-six sex-matched healthy subjects were enrolled in this observational case–control study. A comprehensive ophthalmological evaluation, axial length measurements, and SD-OCT evaluation were performed. The thickness of the retinal layers at the circle centered on the fovea (1 mm in diameter) and the average of the foveal and parafoveal zones at 2 and 3 mm in diameter were evaluated, together with retinal volume and the peripapillary retinal nerve fiber layer (RNFL). Concerning the thicknesses of the retinal layers in each analyzed region, no statistically significant differences were found. The same results were obtained for the total volume. Regarding peripapillary RNFL, the celiac patients showed slightly thicker values than the healthy controls, except for temporal and nasal-inferior quadrants, with no statistically significant differences. All the analyzed parameters were similar for the celiac patients and the healthy individuals. This could be related either to the non-involvement of the retinal layers in celiac disease pathophysiology or to the gluten-free diet effect.

## 1. Introduction

Celiac disease is an immune-mediated, chronic, inflammatory, and systemic illness [1] characterized by the formation of autoantibodies against tissue transglutaminase, which are triggered by gluten and gluten-like proteins in genetically susceptible subjects [2].

Classic celiac disease presents malabsorption, failure to thrive, and diarrhea. At the same time, more subtle presentations such as latent, potential, oligosymptomatic, and extraintestinal signs related to otologic, dental, neurological, dermatological, and musculoskeletal symptoms may be less prevalent [3]. However, individuals are in danger of long-term complications if undetected extraintestinal manifestations are not addressed [4,5].

Among these extraintestinal findings, ocular manifestations due to celiac disease are of great concern because of the direct effect of visual function and ocular comfort on the quality of life [6,7].

The presence of circulating immune complexes or autoantibodies in ocular tissues, cross-reactivity of cell antigenic epitopes, vitamin deficiencies, and immunogenetic factors might all play a role in ocular involvement, especially for all the vascularized components of the eye [6].

In fact, the choroid of celiac patients appears thicker than healthy controls [8,9]. In particular, De Bernardo et al. [9] not only confirmed a thicker choroid in celiac patients [8], but analyzing the choroidal vascularity index in these patients, found no statistical differences between celiac patients and healthy controls. However, celiac patients showed all the choroidal areas to be larger in a significant way than the healthy group. Thus, De Bernardo et al. supposed a proportional increase in both the vascular and stromal components, that may be linked to the inflammatory and autoimmune responses related to celiac disease pathophysiology [9]. On the other hand, anterior eye segment changes due to celiac disease are still unclear [10,11,12].

To the best of our knowledge, no studies have been published examining all the retinal layers concerning retinal involvement in celiac disease. Only a few studies evaluated the peripapillary retinal nerve fiber layer (RNFL), showing no consensus in children and adults [11,12,13,14]. In addition, one study also evaluated the ganglion cell complex (GCC) in a pediatric population, finding no statistical difference between celiac patients and healthy controls [14].

For these reasons, together with the disease’s autoimmune and inflammatory nature and the presence of the superficial and deep capillary plexuses among the retinal layers, this study aims to look for possible signs of retinal involvement, utilizing spectral-domain optical coherence tomography (SD-OCT), that could be utilized as biomarkers for this disease.

## 2. Materials and Methods

### 2.1. Patient Selection

Adult subjects with a diagnosis of celiac disease, evaluated at the Celiac Disease Center at the Department of Medicine, Surgery, and Dentistry of the University of Salerno between September 2019 and March 2020, and a control group of sex-matched healthy subjects were included in this observational case–control study.

Diagnosis of celiac disease was confirmed by intestinal biopsy and serology, regardless of the time of diagnosis. Following the diagnosis, all the celiac patients were placed on a gluten-free diet. Regarding control subjects, they had at least one negative-specific serology for celiac disease and no diagnosis of any gastrointestinal diseases.

Subjects younger than 18 years of age or with systemic and ocular diseases, or patients who underwent other ophthalmic surgical procedures which could affect the eye [15,16,17,18], were excluded from this study.

According to the Declaration of Helsinki’s ethical principles, all participants were informed about the study’s purpose and written informed consent was acquired. Institutional Review Board approval was also obtained from the ComEtico Campania Sud (CECS), prot. n°16544. 

### 2.2. Clinical Examination and OCT Analysis

A comprehensive ophthalmological evaluation, including clinical history to identify possible exclusion criteria, slit-lamp examination, Snellen best-corrected visual acuity, axial length (AL) measurements with IOLMaster (Carl Zeiss Meditec AG, Jena, Germany, version 5.4.4.0006), and SD-OCT evaluation (Spectralis; Heidelberg Engineering; Heidelberg, Germany, version 6.0), was performed.

All participants were examined between 2:00 p.m. and 3:00 p.m., without pupil dilation. For each participant, only the right eye was evaluated [19].

A horizontal 30° volume OCT B-scan centered on the fovea was obtained for all examined eyes. Using the device’s built-in software (Heidelberg Eye Explorer HEYEX; Heidelberg Engineering), the segmentation of the retinal layers was obtained.

Poor-quality images with a signal-to-noise score lower than 20 decibels were excluded.

**Figure 1:**
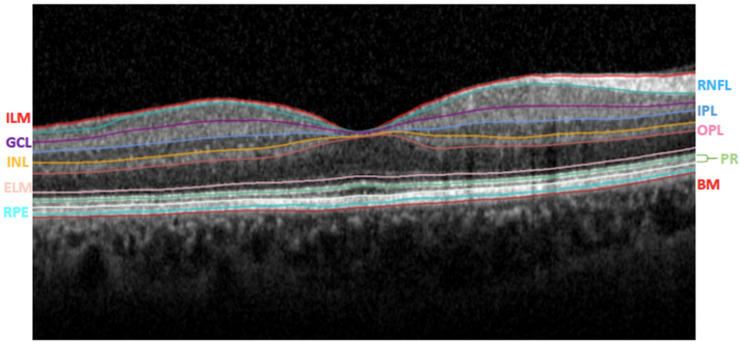
Segmentation of the retinal layers using the instrument’s automatic algorithm. ILM: internal limiting membrane; RNFL: retinal nerve fiber layer; GCL: ganglion cell layer; IPL: inner plexiform layer; INL: inner nuclear layer; OPL: outer plexiform layer; ELM: external limiting membrane; PR: photoreceptor layers; RPE: retinal pigment epithelium; BM: Bruch’s membrane.

To study the 10 retinal layers, eleven optical interfaces were obtained (Figure 1) [20].

**Figure 2:**
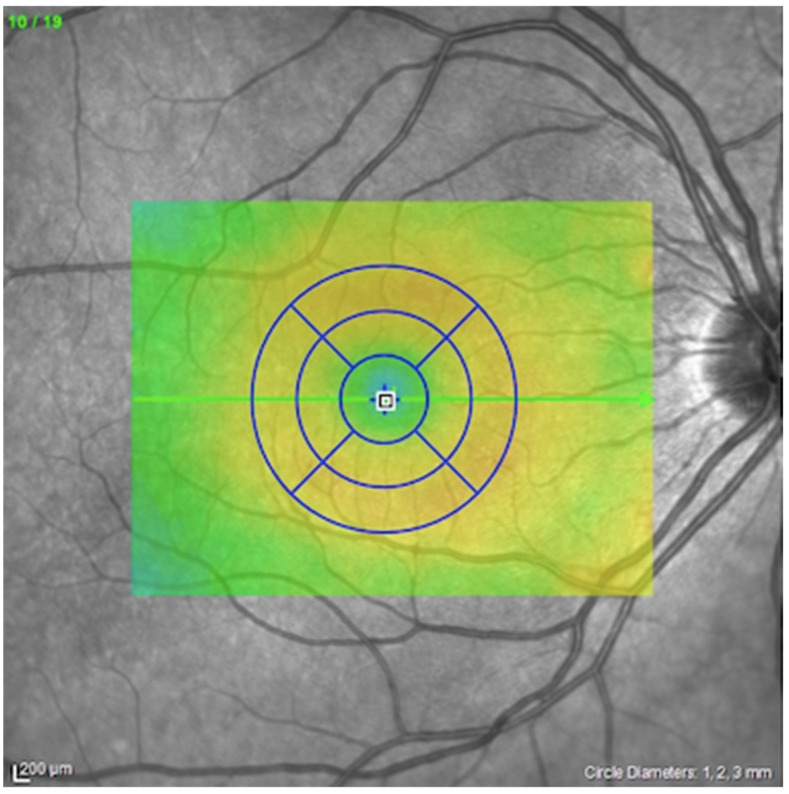
Early Treatment Diabetic Retinopathy Study grid utilized for the retinal analysis.

In addition, utilizing the standard Early Treatment Diabetic Retinopathy Study (ETDRS) grid, the thickness of the retinal layers at the circle centered on the fovea (1 mm in diameter), the average of the 5 foveal and parafoveal zones (2 mm in diameter), and the average of the 9 foveal and parafoveal zones (3 mm in diameter) were evaluated (Figure 2).

For all the analyzed regions (1, 2, and 3 mm diameter), the values of the total thickness (total retina), photoreceptor (PHR) layer, retinal pigment epithelium (RPE), outer nuclear layer (ONL), outer plexiform layer (OPL), the inner retinal layer (IRL), and the GCC thickness were collected. IRL includes the sum of RNFL, GCL, IPL, and the inner nuclear layer (INL), while GCC is composed of RNFL, GCL, and IPL. However, the thickness value for all these layers was also evaluated individually in each studied region of the ETDRS grid.

Moreover, the device’s built-in software automatically calculated the total volume at 3 mm diameter for each retinal layer.

Concerning peripapillary RNFL, the optic nerve head protocol of the device generates an RNFL thickness map from which RNFL thickness is measured along a circle 3.45 mm in diameter centered on the optic disc. The average RNFL thickness of the seven quadrants (global average, temporal, temporal-superior, nasal-superior, nasal, nasal-inferior, and temporal-inferior) was measured for all patients.

### 2.3. Statistical Analysis

All data were analyzed with GraphPad Prism 8 (GraphPad Software, LLC, version 8.4.3). Kolmogorov–Smirnov test was performed to assess normal distribution (*p* > 0.05) for all data.

To compare the different parameters of the two groups, the two-tailed Mann– Whitney U test for not normal-distributed data and the two-tailed independent samples Student *t*-test for normal-distributed data were used. Furthermore, the correlation between the years of gluten-free diet adherence and the total retinal thickness in each analyzed region was also evaluated using the Spearman correlation test. *p* values less than 0.05 were considered statistically significant.

The sample size was determined by maximizing the statistical power. The analysis was performed using G*Power software (version 3.1.9.4) [21]. A difference between two independent means (two groups) was computed. Input data were the following: α was set at 0.05; 1-β was set at 0.81; allocation ratio N2/N1 was set at 1; and the effect size was set as a medium at around 0.5. Results were the following: non-centrality parameter δ = 2.872; critical t = 1.978; Df = 130; sample size group 1 = 66; sample size group 2 = 66; actual power = 0.814; and total sample size = 132.

## 3. Results

Sixty-six patients with celiac disease (nineteen males) and sixty-six sex-matched healthy subjects were enrolled. The mean disease duration of the celiac patients was 9.1 ± 8.8 years (range: 0–41 years). None of the celiac patients included in this study presented previous ocular complications due to celiac disease.

The mean age of the celiac patients was 40.3 ± 11.6 years (range: 18–66 years), while the mean age of the healthy subjects was 39.9 ± 14.2 (range: 23–69 years), with no statistically significant difference between the two groups (*p* = 0.75).

The mean AL of the celiac patients was 23.6 ± 1.0 mm (range: 21.7–26.1 mm), while the mean AL of the healthy subjects was 23.9 ± 1.2 mm (range: 20.7–27.5 mm), with no statistically significant difference between the two groups (*p* = 0.15).

Concerning the thicknesses of the retinal layers at each analyzed region of the ETDRS grid, no statistically significant differences were found between the celiac patients and the healthy subjects, as shown in Table 1, Table 2 and Table 3. However, celiac patients showed slightly thicker retinal layers than healthy subjects, except for INL at 1 mm diameter (Table 1); ONL, INL, and GCL at 2 mm diameter (Table 2); and ONL, GCL, RNFL, and GCC at 3 mm diameter (Table 3).

By comparing the total volume, no statistically significant differences were found as well, as summarized in Table 4.

Regarding peripapillary RNFL, the celiac patients showed slightly thicker values than the healthy controls, except for temporal and nasal-inferior quadrants. Nonetheless, no statistically significant difference for these parameters was found, as shown in Table 5.

Considering the correlation between the years of gluten-free diet adherence and the total retinal thickness, no statistically significant correlation was found at 1 mm (*p* = 0.07; r = −0.23), at 2 mm (*p* = 0.15; r = −0.18), and at 3 mm (*p* = 0.53; r = −0.08).

## 4. Discussion

Celiac disease is a systemic autoimmune disease that primarily affects the small intestine, although it could also present extraintestinal symptoms [22]. The eye is undoubtedly one of the disease’s target organs, with dry eye, cataracts, central retinal vein occlusion, neuro-ophthalmic symptoms, night blindness, uveitis, and thyroid-associated orbitopathy all possible [23].

Considering all these possible ocular complications, an in vivo OCT analysis of the retinal layers and peripapillary RNFL trying to find possible diagnostic signs of ocular involvement in celiac disease might be helpful and of interest.

To the best of our knowledge, the present study is the first one comparing all the retinal layers and the largest one comparing peripapillary RNFL of celiac patients to a healthy control group, to highlight potential differences between the two study groups that could be explained by the underlying pathogenetic mechanisms of celiac disease.

In the present study, celiac patients showed slight diffuse thickening of almost all the retinal layers and peripapillary RNFL, with no statistically significant differences in any of the analyzed parameters.

Concerning the peripapillary RNFL, few previously published papers have addressed this issue without reaching any agreement in the results [11,12,13,14]. Our results confirmed, in adults, the findings obtained by Dereci et al. [14], who, when evaluating both peripapillary RNFL and GCC in 86 eyes of 43 children, found no significant statistical differences between celiac children and healthy controls.

On the other hand, Karatepe Hashas et al. [11] evaluated peripapillary RNFL of 31 celiac children and 34 healthy controls using SD-OCT imaging of both eyes, observing a significant overall thinning of the RNFL in celiac patients. The authors hypothesized that this finding might be attributable to autoantibodies with an affinity to retinal nerve tissue, and they also suggested further pathophysiological studies in order to verify their hypothesis.

The same hypothesis was supported by Hazar et al. [12] who, appraising peripapillary RNFL of 58 eyes of 31 celiac adults and 50 eyes of 25 healthy individuals using SD-OCT, showed a significant thinning of superior RNFL, but a significant thickening of nasal RNFL in celiac patients. Furthermore, the authors found a significant positive correlation between tissue transglutaminase autoantibody levels and the thinning of the superior RNFL, supposing an autoantibody affinity to retinal nerve tissue, as Karatepe Hashas et al. [11] found. However, no explanation on the nasal RNFL thickening was given [12].

On the other hand, Dönmez Gün et al. [13] analyzed 72 eyes of 36 celiac adults and 70 eyes of 35 age- and sex-matched healthy controls with a SD-OCT, showing an overall thinning of peripapillary RNFL in celiac patients, but without statistically significant differences between the two study groups.

Several explanations could be adduced to elucidate some differences between the previous studies [11,12,13,14] and the present one.

First, the current study utilized the largest sample size, which was determined using a power calculation assessment [21]. As a result, previous papers [11,12,13,14] may have yielded different results that contradicted one another due to small and insignificant sample sizes.

Furthermore, the present study examined just one eye per participant, whereas all prior studies [11,12,13,14] examined both eyes in some individuals and only one eye in others. According to McAlinden et al. [24,25], this might lead to statistical bias, affecting the results.

However, in the present study, no significant modification in the thicknesses of all retinal layers, especially for GCC layers, was found, confirming the findings by Dereci et al. [14]. This could make neural tissue involvement a more complicated issue [26].

The GCC is the sum of the three innermost layers: the RNFL, the ganglion cell layer (GCL), and the inner plexiform layer (IPL) [26]. The thickness of the GCC layers could be measured using SD-OCT to assess early signs of systemic and autoimmune disorders [27,28]. The thickness of the GCC layers was demonstrated to be reduced in some pathological conditions, such as systemic lupus erythematosus, Behçet’s disease, obesity, and multiple sclerosis due to the impact of autoinflammatory disorders and metabolic stress [29,30].

According to the assumptions by Karatepe Hashas et al. [11] and Hazar et al. [12], the autoantibodies would cause a decrease in RNFL, GCL, and IPL, but these retinal layers seem to not be reached by these antibodies [29], even if further pathophysiological studies are needed to better understand this issue. Nevertheless, they can be affected by inflammatory processes, as it happens in the case of systemic lupus erythematosus, Behçet’s disease, and multiple sclerosis. Several studies reported decreased thicknesses of the GCC layers, demonstrating that the inflammatory effects of these diseases directly influence neural tissue [29,30].

The present study’s results indicate that celiac disease’s inflammatory and autoimmune processes could not involve the retinal layers directly. However, this finding may also be explained by the gluten-free diet adherence of all analyzed celiac patients, possibly determining a remission of any retinal changes or a decrease in the inflammatory effects of the disease [31].

The fact that the patients were on a gluten-free diet could represent a limitation of the present study. Further studies in naïve celiac patients, comparing the effects of a gluten-free diet versus a regular diet, would be needed to understand better the retinal baseline status of such subjects and its possible changes over time.

## 5. Conclusions

In conclusion, retinal layer thicknesses, volumes, and peripapillary RNFL were similar in the celiac patients and the healthy individuals. The reason for these results could be due to either the non-involvement of the retinal layers in celiac disease or the gluten-free diet effect. However, the results of this study cannot omit a routine ophthalmological examination for these patients due to the association between celiac disease and other ocular disorders [4,5,6,7].

## Figures and Tables

**Table 1:** Comparison of average retinal layer thicknesses (μm) between celiac patients and healthy subjects at 1 mm diameter of ETDRS grid on OCT.

	Celiac Patients 19 Males–47 Females		Healthy Controls 19 Males–47 Females		*p*-Value
	Mean ± SD (Range)	Median (IQ Range)	Mean ± SD (Range)	Median (IQ Range)	
PHR (μm)	88.2 ± 3.3 (82.0–99.0)	88.0 (86.0–90.0)	87.9 ± 3.4 (81.0–95.0)	87.0 (85.8–90.3)	^a^ 0.67
IRL (μm)	182.6 ± 17.7 (148.0–221.0)	181.5 (171.8–196.0)	180.7 ± 19.8 (137.0–237.0)	179.0 (167.0–195.0)	^b^ 0.57
RPE (μm)	16.1 ± 1.5 (13.0–19.0)	16.0 (15.0–17.0)	15.9 ± 1.8 (12.0–19.0)	16.0 (15.0–17.0)	^a^ 0.72
ONL (μm)	92.7 ± 10.2 (65.0–117.0)	92.5 (87.0–100.3)	92.2 ± 9.9 (64.0–115.0)	92.0 (86.8–99.0)	^b^ 0.78
OPL (μm)	26.2 ± 5.2 (17.0–41.0)	26.0 (22.0–29.0)	25.6 ± 5.8 (16.0–43.0)	25.0 (21.8–29.0)	^a^ 0.55
INL (μm)	18.3 ± 5.1 (9.0–34.0)	18.0 (14.0–21.0)	19.1 ± 5.6 (11.0–37.0)	19.0 (14.8–23.0)	^a^ 0.51
IPL (μm)	20.3 ± 3.6 (13.0–29.0)	20.0 (17.0–23.0)	19.5 ± 3.4 (13.0–31.0)	19.0 (17.0–22.0)	^a^ 0.39
GCL (μm)	14.6 ± 4.3 (8.0–25.0)	14.0 (12.0–17.0)	13.9 ± 3.9 (7.0–30.0)	13.0 (11.8–16.0)	^a^ 0.40
RNFL (μm)	12.2 ± 2.0 (7.0–17.0)	12.0 (11.0–14.0)	11.9 ± 2.4 (7.0–19.0)	12.0 (10.0–13.0)	^a^ 0.49
GCC (μm)	46.8 ± 9.3 (28.0–71.0)	46.0 (40.0–53.3)	45.3 ± 9.0 (27.0–77.0)	44.5 (40.0–50.3)	^b^ 0.37
TOTAL RETINA (μm)	270.8 ± 18.2 (235.0–308.0)	270.0 (257.5–284.3)	268.6 ± 20.2 (228.0–328.0)	266.0 (254.5–283.3)	^b^ 0.52

^a^ Mann Whitney U test; ^b^ Student t-test unpaired. SD: Standard Deviation; IQ: Interquartile; PHR: Photoreceptors; IRL: Inner Retinal Layer; RPE: Retinal Pigment Epithelium; ONL: Outer Nuclear Layer; OPL: Outer Plexiform Layer; INL: Inner Nuclear Layer; IPL: Inner Plexiform Layer; GCL: Ganglion Cell Layer; RNFL: Retinal Nerve Fiber Layer; GCC: Ganglion Cell Complex.

**Table 2:** Comparison of average retinal layer thicknesses (μm) between celiac patients and healthy subjects at 2 mm diameter of ETDRS grid on OCT.

	Celiac Patients 19 Males–47 Females		Healthy Controls 19 Males–47 Females		*p*-Value
	Mean ± SD (Range)	Median (IQ Range)	Mean ± SD (Range)	Median (IQ Range)	
PHR (μm)	84.0 ± 3.4 (77.6–95.6)	83.8 (81.8–85.8)	83.6 ± 2.8 (77.4–89.6)	83.8 (81.8–85.8)	^a^ 0.48
IRL (μm)	236.2 ± 13.2 (214.2–272.2)	234.4 (226.6–245.6)	235.3 ± 16.5 (193.8–267.6)	234.8 (223.0–250.6)	^a^ 0.72
RPE (μm)	15.6 ± 1.5 (12.6–19.8)	15.6 (14.8–16.8)	15.5 ± 1.5 (11.8–18.8)	15.4 (14.2–16.6)	^a^ 0.24
ONL (μm)	76.9 ± 9.7 (59.0–103.0)	75.2 (70.9–84.3)	77.0 ± 9.5 (55.0–100.0)	77.7 (70.6–84.4)	^b^ 0.79
OPL (μm)	34.6 ± 5.3 (24.8–48.6)	33.9 (31.2–38.5)	33.4 ± 5.6 (24.8–46.0)	31.9 (29.3–37.8)	^b^ 0.12
INL (μm)	34.1 ± 4.0 (24.8–44.8)	33.5 (31.4–37.3)	34.5 ± 4.1 (27.8–44.2)	34.4 (30.8–37.4)	^a^ 0.61
IPL (μm)	35.7 ± 3.3 (29.2–42.8)	36.2 (34.2–37.9)	35.5 ± 3.7 (27.4–43.6)	35.2 (32.8–38.6)	^a^ 0.76
GCL (μm)	39.3 ± 5.2 (27.8–51.4)	39.4 (35.3–43.5)	39.4 ± 5.2 (28.8–50.2)	39.1 (35.6–43.2)	^a^ 0.97
RNFL (μm)	16.0 ± 0.9 (13.6–18.6)	15.8 (15.4–16.6)	15.9 ± 1.2 (13.6–20.0)	15.6 (15.0–16.6)	^b^ 0.50
GCC (μm)	91.0 ± 8.5 (74.4–112.2)	91.7 (84.9–97.1)	90.8 ± 9.3 (72.2–110.2)	90.2 (84.3–97.6)	^a^ 0.90
TOTAL RETINA (μm)	320.3 ± 14.6 (298.4–358.6)	318.9 (308.2–330.9)	318.9 ± 17.0 (272.0–354.8)	318.4 (307.9–329.9)	^a^ 0.62

^a^ Student *t*-test unpaired; ^b^ Mann Whitney U test. SD: Standard Deviation; IQ: Interquartile; PHR: Photoreceptors; IRL: Inner Retinal Layer; RPE: Retinal Pigment Epithelium; ONL: Outer Nuclear Layer; OPL: Outer Plexiform Layer; INL: Inner Nuclear Layer; IPL: Inner Plexiform Layer; GCL: Ganglion Cell Layer; RNFL: Retinal Nerve Fiber Layer; GCC: Ganglion Cell Complex.

**Table 3:** Comparison of average retinal layer thicknesses (μm) between celiac patients and healthy subjects at 3 mm diameter of ETDRS grid on OCT.

	Celiac Patients 19 Males–47 Females		Healthy Controls 19 Males–47 Females		*p*-Value
	Mean ± SD (Range)	Median (IQ Range)	Mean ± SD (Range)	Median (IQ Range)	
PHR (μm)	82.1 ± 3.1 (75.9–91.3)	81.7 (80.5–84.3)	81.7 ± 2.5 (75.8–86.8)	81.7 (80.2–83.6)	^a^ 0.44
IRL (μm)	248.7 ± 12.4 (227.2–284.8)	246.7 (238.1–256.4)	248.3 ± 14.2 (211.6–277.4)	248.7 (237.1–258.6)	^a^ 0.86
RPE (μm)	14.9 ± 1.4 (12.2–19.1)	14.8 (13.8–16.0)	14.6 ± 1.3 (11.4–17.6)	14.7 (13.6–15.6)	^a^ 0.17
ONL (μm)	72.7 ± 9.1 (56.2–96.6)	70.9 (67.0–78.0)	72.9 ± 8.7 (51.9–94.6)	73.8 (66.5–79.2)	^b^ 0.76
OPL (μm)	33.8 ± 4.5 (25.8–47.1)	33.8 (30.4–37.1)	33.1 ± 4.8 (25.8–45.6)	31.9 (29.3–37.0)	^b^ 0.30
INL (μm)	37.5 ± 3.2 (30.4–46.3)	37.1 (35.3–39.8)	37.5 ± 3.5 (31.9–45.8)	37.3 (34.9–39.8)	^a^ 0.98
IPL (μm)	39.0 ± 2.7 (32.4–45.6)	39.3 (37.6–40.4)	38.9 ± 3.0 (31.6–46.6)	38.7 (36.8–41.1)	^a^ 0.88
GCL (μm)	46.5 ± 4.2 (36.3–55.7)	46.3 (43.2–49.8)	46.6 ± 4.3 (36.6–55.8)	45.9 (43.4–50.2)	^a^ 0.89
RNFL (μm)	19.5 ± 1.5 (16.6–22.4)	19.6 (18.6–20.6)	19.6 ± 1.7 (16.2–24.4)	19.3 (18.2–20.6)	^a^ 0.88
GCC (μm)	105.0 ± 7.5 (85.7–121.8)	105.2 (100.2–110.6)	105.1 ± 8.2 (87.0–124.3)	104.0 (99.1–112.6)	^a^ 0.96
TOTAL RETINA (μm)	330.9 ± 13.8 (303.3–369.3)	329.7 (318.9–341.1)	330.0 ± 14.7 (289.0–362.1)	330.6 (319.7–338.7)	^a^ 0.74

^a^ Student *t*-test unpaired; ^b^ Mann Whitney U test. SD: Standard Deviation; IQ: Interquartile; PHR: Photoreceptors; IRL: Inner Retinal Layer; RPE: Retinal Pigment Epithelium; ONL: Outer Nuclear Layer; OPL: Outer Plexiform Layer; INL: Inner Nuclear Layer; IPL: Inner Plexiform Layer; GCL: Ganglion Cell Layer; RNFL: Retinal Nerve Fiber Layer; GCC: Ganglion Cell Complex.

**Table 4:** Comparison of total volume (mm^3^) between celiac patients and healthy subjects of the analyzed OCT scan.

	Celiac Patients 19 Males–47 Females		Healthy Controls 19 Males–47 Females		*p*-Value
	Mean ± SD (Range)	Median (IQ Range)	Mean ± SD (Range)	Median (IQ Range)	
PHR (mm^3^)	0.58 ± 0.02 (0.54–0.64)	0.58 (0.57–0.59)	0.58 ± 0.02 (0.53–0.61)	0.58 (0.57–0.59)	^a^ 0.56
IRL (mm^3^)	1.77 ± 0.09 (1.61–2.02)	1.77 (1.70–1.83)	1.77 ± 0.10 (1.52–1.97)	1.77 (1.69–1.82)	^a^ 0.97
RPE (mm^3^)	0.10 ± 0.01 (0.09–0.13)	0.10 (0.10–0.11)	0.10 ± 0.01 (0.08–0.12)	0.10 (0.10–0.11)	^a^ 0.85
ONL (mm^3^)	0.51 ± 0.06 (0.39–0.67)	0.50 (0.47–0.55)	0.51 ± 0.06 (0.37–0.66)	0.52 (0.47–0.55)	^a^ 0.74
OPL (mm^3^)	0.24 ± 0.03 (0.18–0.33)	0.24 (0.21–0.26)	0.23 ± 0.03 (0.18–0.32)	0.22 (0.21–0.26)	^a^ 0.46
INL (mm^3^)	0.27 ± 0.02 (0.22–0.33)	0.27 (0.25–0.28)	0.27 ± 0.02 (0.23–0.33)	0.27 (0.25–0.28)	^a^ 0.87
IPL (mm^3^)	0.28 ± 0.02 (0.23–0.33)	0.28 (0.27–0.29)	0.28 ± 0.02 (0.23–0.33)	0.28 (0.26–0.29)	^a^ 0.60
GCL (mm^3^)	0.34 ± 0.03 (0.26–0.40)	0.34 (0.32–0.36)	0.34 ± 0.03 (0.27–0.40)	0.33 (0.32–0.36)	^a^ 0.97
RNFL (mm^3^)	0.14 ± 0.01 (0.12–0.17)	0.14 (0.14–0.15)	0.14 ± 0.01 (0.12–0.18)	0.14 (0.13–0.15)	^a^ 0.95
GCC (mm^3^)	0.76 ± 0.06 (0.62–0.90)	0.75 (0.71–0.80)	0.76 ± 0.06 (0.67–0.88)	0.75 (0.71–0.81)	^a^ 0.98
TOTAL RETINA (mm^3^)	2.35 ± 0.10 (2.14–2.62)	2.34 (2.27–2.42)	2.34 ± 0.10 (2.07–2.57)	2.35 (2.27–2.40)	^b^ 0.81

^a^ Mann Whitney U test; ^b^ Student *t*-test unpaired. SD: Standard Deviation; IQ: Interquartile; PHR: Photoreceptors; IRL: Inner Retinal Layer; RPE: Retinal Pigment Epithelium; ONL: Outer Nuclear Layer; OPL: Outer Plexiform Layer; INL: Inner Nuclear Layer; IPL: Inner Plexiform Layer; GCL: Ganglion Cell Layer; RNFL: Retinal Nerve Fiber Layer; GCC: Ganglion Cell Complex.

**Table 5:** Comparison of peripapillary RNFL thicknesses (μm) between celiac patients and healthy subjects.

	Celiac Patients 19 Males–47 Females		Healthy Controls 19 Males–47 Females		*p*-Value
	Mean ± SD (Range)	Median (IQ Range)	Mean ± SD (Range)	Median (IQ Range)	
G (μm)	100.3 ± 11.5 (62.0–127.0)	102.0 (93.0–110.0)	99.5 ± 10.1 (72.0–127.0)	99.0 (94.0–104.3)	^a^ 0.69
T (μm)	76.2 ± 13.1 (48.0–117.0)	75.5 (67.0–82.0)	79.4 ± 13.8 (53.0–128.0)	78.0 (69.8–90.0)	^b^ 0.13
TS (μm)	133.6 ± 24.3 (42.0–190.0)	137.5 (120.0–145.5)	131.9 ± 17.8 (96.0–170.0)	133.0 (117.0–144.3)	^a^ 0.64
NS (μm)	112.6 ± 23.0 (23.0–168.0)	113.0 (102.8–126.5)	105.7 ± 24.3 (39.0–171.0)	106.0 (93.8–119.0)	^a^ 0.09
N (μm)	76.1 ± 14.3 (43.0–115.0)	78.0 (65.8–86.0)	74.0 ± 15.3 (40.0–123.0)	70.5 (63.8–83.3)	^b^ 0.23
NI (μm)	107.4 ± 26.8 (48.0–187.0)	106.5 (88.3–124.5)	111.0 ± 28.2 (53.0–198.0)	107.5 (88.8–125.8)	^a^ 0.46
TI (μm)	144.0 ± 20.9 (84.0–185.0)	142.5 (134.0–159.5)	140.9 ± 20.9 (88.0–186.0)	140.0 (129.0–157.0)	^a^ 0.39

^a^ Student *t*-test unpaired; ^b^ Mann Whitney U test. SD: Standard Deviation; IQ: Interquartile; G: Global average; T: Temporal; TS: Temporal-Superior; NS: Nasal-Superior; N: Nasal; NI: Nasal-Inferior; TI: Temporal-Inferior.

## Data Availability

The data presented in this study are available on request from the corresponding author.
